# Effects of Recommended Supplementation and Mediterranean Diet Adherence on Post-Metabolic Bariatric Surgery Outcomes

**DOI:** 10.3390/biomedicines14030513

**Published:** 2026-02-26

**Authors:** Jan Dębski, Anna Sabuć, Antonina Spalińska, Józef Przybyłowski, Klaudia Skibiak, Maria Czerwińska, Joanna Dzedzej, Emilia Talarek, Amelia Gierula, Krzysztof Wyszomirski, Maciej Walędziak, Anna Różańska-Walędziak

**Affiliations:** 1 Medical Faculty, Collegium Medicum, Cardinal Stefan Wyszynski University in Warsaw, 01-938 Warsaw, Poland; jdebski@student.uksw.edu.pl (J.D.); a.sabuc@student.uksw.edu.pl (A.S.); j.przybylowski@student.uksw.edu.pl (J.P.); k.skibiak@student.uksw.edu.pl (K.S.); mariaczerwinska@student.uksw.edu.pl (M.C.); j.dzedzej@student.uksw.edu.pl (J.D.); emiliatalarek1@wp.pl (E.T.); amelia.gierula1@gmail.com (A.G.); 2 Department of Biostatistics and Research Methodology, Faculty of Medicine, Collegium Medicum, Cardinal Stefan Wyszynski University in Warsaw, 01-938 Warsaw, Poland; a.slubowska@uksw.edu.pl; 3 Department of Human Physiology and Pathophysiology, Faculty of Medicine, Collegium Medicum, Cardinal Stefan Wyszynski University in Warsaw, 01-938 Warsaw, Poland; kj.wyszomirski@gmail.com; 4 Department of General, Oncological, Metabolic and Thoracic Surgery, Military Institute of Medicine, Szaserów 128 St., 04-141 Warsaw, Poland; maciej.waledziak@gmail.com

**Keywords:** bariatric surgery, mediterranean diet, nutritional deficiencies, iron deficiency, protein status, excess weight loss, postoperative supplementation

## Abstract

**Background/Objectives:** Metabolic bariatric surgery is an effective treatment for severe obesity but is associated with an increased risk of postoperative nutritional deficiencies. This study aimed to assess the impact of adherence to the Mediterranean diet on selected laboratory parameters and excess weight loss after bariatric surgery. **Methods:** Eighty adults with obesity were evaluated before and 6 months after surgery. Based on dietary questionnaires, patients were classified as adhering to the Mediterranean diet (*n* = 32) or not adhering (*n* = 48). Laboratory parameters, including vitamin D, vitamin B12, folic acid, iron, ferritin, calcium, hemoglobin, and total protein, were assessed pre- and postoperatively. Excess weight loss percentage (EWL%) was calculated using standard methodology. **Results:** Postoperatively, vitamin D and total protein levels increased in both groups, with median increases of 9.45 ng/mL and 1.1 g/dL, respectively. A significant increase in iron concentration was observed only in Mediterranean diet adherents (median +24 µg/dL), while a decrease was noted in non-adherent patients (−4 µg/dL). Iron deficiency and iron-deficiency anemia occurred exclusively in the non-Mediterranean diet group (10.4% vs. 0%). Median EWL% was higher in Mediterranean diet adherents (44% vs. 31%), although the difference was not statistically significant. **Conclusions:** Adherence to the Mediterranean diet may reduce the risk of iron deficiency after bariatric surgery, whereas total protein concentration alone appears insufficient for assessing nutritional status or weight loss effectiveness.

## 1. Introduction

According to the definition of the World Health Organization, obesity is the excessive accumulation of fat tissue that poses a health risk [1]. This chronic disease is determined by a body mass index (BMI) of 30 kg/m^2^, and its prevalence can be described as a pandemic [2,3]. Although it is associated with significant weight gain, most patients remain malnourished because of a high-carbohydrate diet, metabolic imbalance and the chronic inflammation that occurs in obesity [4,5]. Malnutrition manifests itself through deficiencies, especially vitamin D3, vitamin B12, folic acid, iron, calcium and protein, which may cause serious disorders [4,5].

Metabolic bariatric surgery is the most effective method of treating advanced obesity, which provides long-term results [6]. Patients qualified for surgery must have a Body Mass Index (BMI) ≥ 40 kg/m^2^ or a BMI ≥ 35 kg/m^2^ with at least one coexisting disease, such as hypertension, osteoarthritis, obstructive sleep apnea, type 2 diabetes or depression, which may go into remission after weight loss [7,8]. The most popular procedures of this method include laparoscopic sleeve gastrectomy (LSG) and laparoscopic Roux-en-Y gastric bypass (LRYGB) [6]. The effectiveness of this method is measured by the percentage of excess weight loss (EWL%), which expresses the percentage of excess body weight lost [9]. Values of EWL% > 50% are considered effective weight loss during bariatric surgery [9,10].

Unfortunately, this method of treatment also carries the risk of deficiencies in the above-mentioned nutrients [11,12]. This is related to a significantly reduced absorption area of the gastrointestinal tract, resulting from bypassing procedures, and reduced production of gastric acid, which particularly contributes to the development of iron and vitamin B12 deficiency, from restrictive procedures [5]. A 2019 study further found that while both types of procedures contributed to the occurrence of deficiencies, Roux-en-Y gastric bypass was associated with a higher risk of deficiency, which involved bypassing the stomach, duodenum and part of the jejunum [5,11].

To improve the weight loss process and at the same time prevent micronutrient deficiencies after bariatric surgery, the International Federation for the Surgery of Obesity and Metabolic Disorders (IFSO) has developed guidelines that precisely describe both qualifications for surgery and pre- and post-operative care [5,8,13]. In order to eliminate the effects of impaired absorption of nutrients, it is recommended, among others, physical activity, an easily digestible diet, monitoring and supplementation of vitamins D3 and B12, folic acid, iron and calcium after the procedure [5,8,13]. An equally important aspect is also supplementing protein deficiencies after surgery [14]. It plays an important role in weight loss, increasing fat-free mass and body regeneration [5,8,13,15]. Metabolic bariatric surgery and subsequent reducing protein intake may result in a decrease in fat-free body mass, which slows down resting metabolism and inhibits the feeling of satiety, which may potentially lead to reduced weight loss [12,16,17]. Current dietary guidelines recommend intake of 60–80 g per day or 1–1.5 g/kg ideal body weight (IBW) of protein after bariatric surgery [8,13,15]. Despite recommendations, patients often consume protein in small amounts due to food intolerances and post-bariatric changes in the structure of the digestive tract, resulting in reduced protein digestion and absorption [12]. For this reason, additional whey protein supplementation is introduced to overcome the reluctance to eat protein foods in the first year after surgery [8,18]. Additionally, some studies have shown that increased protein intake after surgery resulted in greater weight loss but without greater preservation of fat-free body mass [14,15]. A parameter often used in clinical practice to roughly assess the protein nutritional status is the concentration of total protein [19]. However, many recent studies show that this marker may not be sufficient to determine proper nutrition due to various clinical conditions affecting its values [20,21].

Particularly noteworthy is the well-known Mediterranean diet, which includes fruits and vegetables as desserts, as well as whole grain products, nuts, legumes, fish, seafood and olive oil as a source of fat [22,23]. Eggs, red meat and high-fat dairy products are also consumed, but in small quantities [23,24]. As part of the diet, moderate alcohol consumption is allowed, mainly red wine with meals [23,25]. Many studies have shown its positive effect on weight loss and improvement of BMI, as well as its anti-inflammatory effect, which makes it an effective diet in the treatment of obesity [3]. The Mediterranean diet is also a source of many nutrients such as protein, vitamin D, vitamin B12, folic acid, iron and calcium, deficiencies of which are observed both in obesity and after bariatric surgery [26,27]. Its effectiveness was also demonstrated in an 8-week intervention aimed at assessing the impact of diet on the treatment of obesity in the period before bariatric surgery [26]. The results showed that the use of a diet enriched with protein supplementation contributed to a greater reduction in body fat while maintaining lean body mass [26]. Studies were also carried out to assess the effectiveness of the diet after bariatric surgery, which resulted in a significant decrease in BMI, reduced glucose levels and an improvement in the lipid profile under the influence of the diet [26]. However, the impact of dietary patterns on postoperative nutritional status and weight loss outcomes remains inconsistent across studies [26].

Recent studies show that despite postoperative supplementation, some patients may still experience deficiencies of specific nutrients, especially vitamin D, vitamin B12, folic acid and iron, both in the first months and several years after surgery [7,13,28,29]. The reason for this phenomenon may be insufficient adherence of patients to the recommendations caused by intolerance of supplements, especially iron, as well as the fact that during the procedures, there is a significant reduction in the absorption surface of vitamins and microelements, which contributes to the limited bioavailability of oral preparations [29,30,31,32]. Therefore, additional nutritional solutions, such as adherence to the Mediterranean diet, which is a source of many nutrients [26,27], may potentially support standard post-operative supplementation and contribute to reducing the risk of nutritional deficiencies, as well as being associated with potentially more favorable weight loss outcomes after bariatric surgery [33].

### The Aim of the Study

The aim of this study was to assess the impact of adherence to a Mediterranean diet, used in conjunction with recommended postoperative supplementation, on selected laboratory parameters. Furthermore, the study aimed to assess the impact of adherence to a Mediterranean diet on excess body weight loss, expressed as EWL%. It also included an independent assessment of the relationship between protein nutritional status and recommended postoperative protein supplementation on weight loss outcomes. Additionally, we analyzed whether total protein concentration before and after surgery could serve as a proxy for protein nutritional status and whether its values were associated with the extent of excess body weight loss after surgery.

## 2. Materials and Methods

### 2.1. Questionnaire and Laboratory Tests

The study involved 99 patients from the Department of General, Oncological, Metabolic, and Thoracic Surgery at the Military Institute of Medicine—National Research Institute in Warsaw. They were asked to come to two follow-up visits: first, before surgery, and second, 6 months after surgery, during which they were examined subjectively and physically, had selected laboratory parameters determined and answered the questions included in the original questionnaire. The inclusion criteria for the study and subsequent analysis included: age over 18 years, informed consent, indications for bariatric surgery [7,8] and having a complete set of survey, clinical and laboratory data available both preoperatively and postoperatively, enabling paired analysis. Nineteen patients were disqualified from further analysis due to failure to return for a postoperative follow-up visit to complete questionnaires and undergo clinical and laboratory re-evaluation. The reason for the loss of these observations was the fact that some patients living far from our study center did not return for the second follow-up visit for repeated clinical and laboratory evaluation and to answer the survey questions. The final sample of participants eligible for further analysis included 80 patients who completed study surveys and had laboratory parameters determined both before and after bariatric surgery. Our survey includes research tools such as dietary inquiries. Patients also had their Body Mass Index (BMI) calculated before and after surgery and the percentage of excessive weight loss estimated (EWL%). The survey also included information on the patients’ age, gender, height, weight, education, profession and the type of surgical procedure performed. Participants also had laboratory parameters assessed, including glucose, hemoglobin, glycated hemoglobin, calcium, iron, vitamin D, vitamin B12, folic acid, ferritin, total protein, lipid profile, liver function tests, and red blood cell count and hematocrit, both before and 6 months after surgery.

### 2.2. Diet Groups and Dietary Questionnaires

Bariatric patients were asked about the type and frequency of meals consumed before and 6 months after surgery. Foods listed in the dietary questionnaires included red meat, eggs, fish and seafood, milk and dairy products, poultry, soy and soy products, cereal products, as well as cauliflower, cabbage, and their products. The frequency of meal consumption was measured by responses such as “never,” “once a month or less,” “2–3 times a month,” “1–2 times a week,” “3–4 times a week,” “5–6 times a week,” “1 time a day,” “2–3 times a day,” “4–5 times a day,” and “6 or more times a day”. Patients who answered the questions about their diet: red meat a maximum of “2–3 times a month”, poultry and eggs a maximum of “1–2 times a week”, fish, seafood and cauliflower at least “1–2 times a week” and milk and dairy products, as well as whole grain products at least “5–6 times a week”, in accordance with the current medical literature, were defined as those who follow the Mediterranean diet and assigned to the “On Diet” group, and the others as those who do not follow this diet and assigned to the “Not On Diet” group [3,22,23,24,25]. Data collection on dietary intake was part of a broader clinical assessment of patients after bariatric surgery; however, diet was not the primary variable examined in the project, and this analysis focuses solely on the relationships between self-reported adherence to a Mediterranean diet, nutritional status, and weight loss outcomes.

### 2.3. IFSO Recommendations

All patients who participated in the study were treated according to the recommendations of the International Federation for the Surgery of Obesity and Metabolic Disorders [13]. In the preoperative phase, the guidelines included psychological consultation, monitoring and possible supplementation of nutritional deficiencies, a physical exercise program, and stabilization of the patient in terms of comorbidities associated with obesity [7,8,34].

Immediately after surgery, patients were introduced to a liquid diet lasting 3–7 days with a gradually increased protein intake [35]. Then they were slowly switching to an easily digestible solid diet in which the main energy source was protein [13,35]. This stage lasted up to 4 weeks after surgery [35]. All patients had a recommended protein intake of 60–80 g per day or 1–1.5 g/kg ideal body weight (IBW) [8,13,15]. Additionally, they were provided with whey protein supplementation at a dose of 30 g per day [36].

In order to compensate for the deficiencies, they were additionally required to supplement orally microelements such as: vitamin D at a dose of 3000–6000 IU per day, vitamin B12 at a dose of 350–1000 µg per day, folic acid at a dose of 400–800 µg per day, iron at a dose of 150–200 mg per day and calcium at a dose of 1200–1500 mg per day [8,13]. In the post-bariatric period, patients were under the constant care of a bariatric dietitian and had their laboratory parameters monitored, including nutrients, to supplement deficiencies [8,13,35,36].

### 2.4. Laboratory Norms and Definitions of Deficiencies

Deficiencies of individual nutrients and iron deficiency anemia were determined on the basis of laboratory standards applicable as reference values in the laboratory where the laboratory tests were performed. The reference range for supplemented nutrients is as follows: vitamin D: 20–80 ng/mL, vitamin B12: 191–663 pg/mL, folic acid: 4.5–37.3 ng/mL, iron: 60–180 µg/dL, total calcium: 8.6–10.2 mg/dL and total protein: 6–8 g/dL. For vitamins and trace elements, deficiencies were defined based on values below the lower limit of laboratory norms. Total protein concentration, below the lower limit of normal, was considered a guideline parameter. To assess protein deficiency, we also assessed the presence of clinical symptoms characteristic of this condition, such as peripheral edema [37,38].

The laboratory definition of iron deficiency anemia was established as the following values: hemoglobin below 13 g/dL in men and below 12 g/dL in women, ferritin below 100 ng/mL and iron below 60 µg/dL regardless of gender. A ferritin value below 100 ng/mL has been considered as iron deficiency due to the chronic inflammation occurring in obesity, during which ferritin concentration increases regardless of iron stores [20,39].

### 2.5. EWL%

EWL% was calculated as the ratio of the difference between body weight before and after surgery to the difference between body weight before surgery and the ideal weight corresponding to normal BMI values, multiplied by 100% [9,40].

### 2.6. Statistical Analysis

All statistical analyses were performed using R (R Foundation for Statistical Computing). Continuous variables were assessed for distributional characteristics using visual inspection of histograms and Q–Q plots, supported by the Shapiro–Wilk test. Normally distributed data are presented as mean ± standard deviation (SD), while non-normally distributed variables are presented as median and interquartile range (IQR).

Within-group pre- and postoperative comparisons were performed using paired tests (paired Student’s *t*-test or Wilcoxon signed-rank test, as appropriate). Between-group comparisons of continuous variables were conducted using independent samples *t*-tests or Mann–Whitney U tests, depending on distributional assumptions. Associations between categorical variables were analyzed using Fisher’s exact test due to small subgroup sizes.

For outcomes demonstrating statistically significant between-group differences in unadjusted analyses, additional multivariable linear regression models were performed to evaluate the robustness of these findings. Postoperative values were modeled as dependent variables with adjustment for baseline concentration of the respective parameter and clinically relevant covariates selected a priori based on biological plausibility, including sex and type of surgical procedure. Model assumptions were evaluated using residual diagnostics, including assessment of normality, homoscedasticity, and influence measures. Sensitivity analyses excluding influential observations were conducted when appropriate.

All tests were two-sided, and statistical significance was defined as *p* < 0.05. Given the exploratory nature of the study and multiple outcomes analyzed, findings should be interpreted cautiously and considered hypothesis-generating.

### 2.7. Ethical Considerations

The study was conducted in accordance with internationally accepted ethical principles, including the Declaration of Helsinki and its subsequent revisions. All participants were informed about the objectives and procedures of the study and provided written informed consent prior to inclusion. Data were collected and analyzed in an anonymized manner. The study protocol was reviewed and approved by the Bioethics Committee of the Military Institute of Medicine in Warsaw (approval no. 30/WIM/2021).

### 2.8. Generative Artificial Intelligence Statement

The authors declare that no generative artificial intelligence tools were used in the design of the study, data analysis, interpretation of results, or preparation of the manuscript.

## 3. Results

### 3.1. General Characteristics of the Study Group

The study included 61 women and 19 men aged 22–68 who underwent metabolic bariatric surgery. Among them, 11 people underwent Roux-en-Y gastric bypass and 69 underwent sleeve gastrectomy. Their preoperative Body Mass Index ranged from 31.44 to 78.9 kg/m^2^ and post-bariatric was from 25.40 to 61.23 kg/m^2^. All patients after bariatric surgery complied with the recommended protein, vitamin and micronutrient supplementation [7]. Additionally, all our patients had no peripheral edema or other signs of malnutrition 6 months after surgery [37,38].

### 3.2. Mediterranean Diet Adherence

There were 32 patients who answered the questions about their diet: red meat a maximum of “2–3 times a month”, poultry and eggs a maximum of “1–2 times a week”, fish, seafood and cauliflower at least “1–2 times a week” and milk and dairy products, as well as whole grain products at least “5–6 times a week”, and 48 patients who gave a different answer.

**Figure 1:**
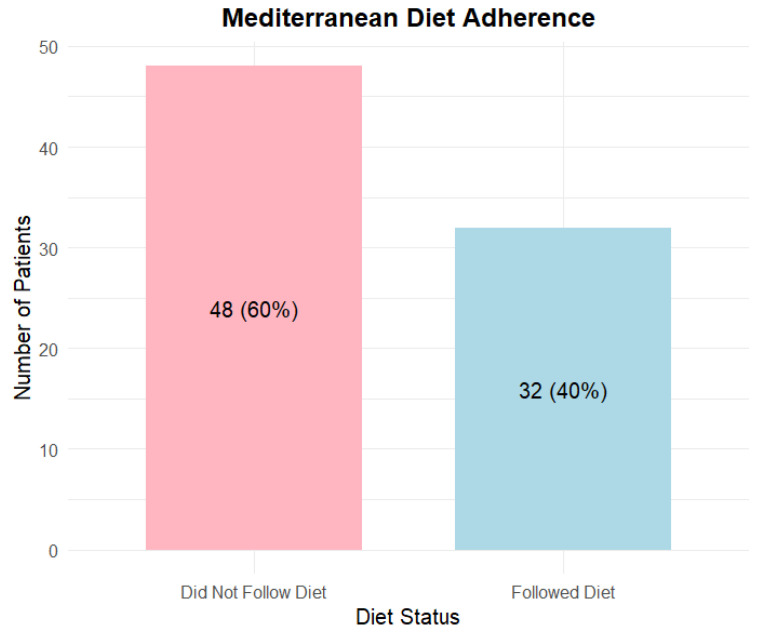
The Mediterranean Diet Adherence in study group. Followed Diet-patients who followed Mediterranean diet; Did Not Follow Diet-patients who did not follow Mediterranean diet.

Based on the dietary questionnaire responses, 32 patients met the Mediterranean diet criteria, while 48 did not follow this diet (Figure 1).

### 3.3. Influence of Mediterranean Diet Adherence and Recommended Supplementation on Laboratory Parameters

#### 3.3.1. Vitamin D

Patients not following the Mediterranean diet had higher preoperative vitamin D levels than those following it (IQR = 18.62–26.83), (Table 1). However, a greater proportion of patients in each dietary group were vitamin D deficient (On Diet: Q3 = 18.55, Not On Diet: Q3 = 18.62), (Table 1). Postoperatively, all patients had normal vitamin D levels, and it was noted that in both dietary groups the increase in vitamin D levels was significant (Not on Diet: Median diff = 8.95, On Diet: Median diff = 10.3, *p* < 0.05), (Table 1), but the mean differences in this parameter before and after surgery between the two interventions were not statistically significant (CI (−4)–3.33, *p* = 0.855), (Table 2).

#### 3.3.2. Vitamin B12

Most patients, regardless of diet, had normal vitamin B12 levels both before and after surgery. Patients following the Mediterranean diet had higher vitamin B12 levels (Median diff = 16.45, *p* < 0.05) (Table 1); however, the differences in vitamin B12 levels between the two dietary groups were not statistically significant (CI (−84.91)–36.59, *p* = 0.431), (Table 2).

#### 3.3.3. Folic Acid

In most patients, folic acid values were within the normal range, and an increase in the concentration of this parameter was observed in both dietary groups; however, no statistical significance was demonstrated between the two nutritional interventions (CI (−2.31)–1.54, *p* = 0.694) (Table 2).

#### 3.3.4. Calcium

Preoperative calcium levels were within normal limits in most patients (Not on Diet: IQR = 9.3–9.8, On Diet: IQR = 9.38–9.72) (Table 1). A slight increase in calcium levels was observed in postoperative patients, both in those following the Mediterranean diet (IQR = 9.6–9.9), (Table 1) and those not following this diet; however, the differences between the two dietary interventions were not statistically significant (CI (−0.29)–0.04, *p* = 0.143), (Table 2).

#### 3.3.5. Total Protein

It was shown that the majority of patients, regardless of the diet used, had protein deficiency before surgery (Not On Diet: IQR = 5.6–6.2, On Diet: IQR = 5.6–6.3) (Table 1). After surgery, all patients had protein concentrations within the reference range; however, the increase in values in both dietary groups was small (Not On Diet: mean = 6.95, On Diet: mean = 6.89), and the differences between the two nutritional interventions were not statistically significant (CI (−0.14)–0.36, *p* = 0.388), (Table 2).

#### 3.3.6. Iron

**Figure 2:**
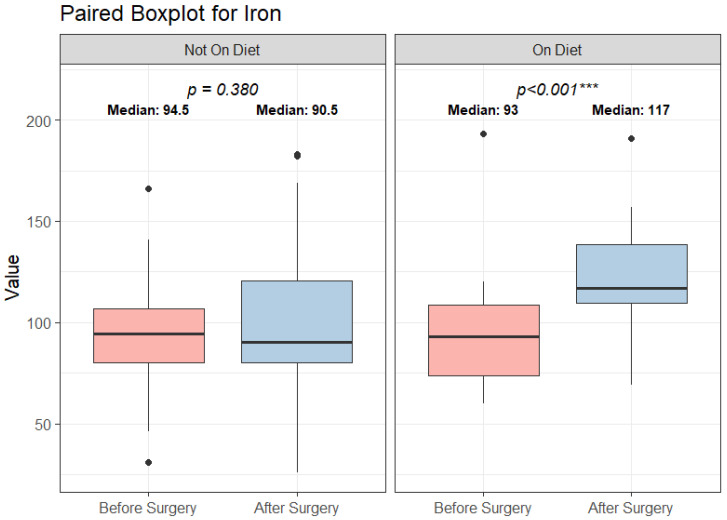
Paired comparison of serum iron levels before and after bariatric surgery stratified by Mediterranean diet adherence. Data are presented as boxplots showing median and interquartile range (IQR), with whiskers representing minimum and maximum values and dots indicating outliers. Pre- and postoperative values were compared using the Wilcoxon signed-rank test. Statistical significance was set at *p* < 0.05. ***—statisticaly significant.

Before surgery, all patients following the Mediterranean diet had normal iron levels, whereas 3 patients not following this diet had both iron deficiency and iron deficiency anemia. Postoperatively, iron levels increased significantly in patients following the Mediterranean diet, and a statistically significant difference was found between the two dietary groups (Median diff = 24.00, CI (−39.04)–(−6.56), *p* < 0.05), (Table 1, Figure 2).

In a multivariable linear regression model with postoperative iron concentration as the dependent variable and adjustment for baseline iron, sex, and type of surgical procedure, adherence to the Mediterranean diet remained independently associated with higher postoperative iron levels (β = 24.89 µg/dL, *p* < 0.001).

Influential observations were assessed using Cook’s distance, with values >0.10 considered indicative of moderate influence. Sensitivity analyses excluding these observations yielded comparable results (β = 24.89 µg/dL, *p* < 0.001), indicating that the association between Mediterranean diet adherence and postoperative iron levels was not driven by influential cases.

Additionally, 5 patients not following the Mediterranean diet had iron deficiency. Among them, four had iron deficiency anemia (Figure 2).

#### 3.3.7. Ferritin and Hemoglobin

**Figure 3:**
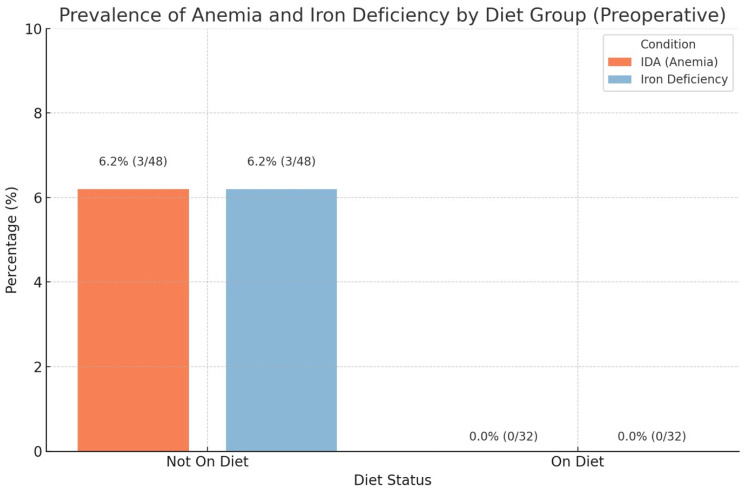
Preoperative prevalence of anemia and iron deficiency in both diet groups. Bars represent the proportion of patients with anemia and iron deficiency in each diet group. Values above bars are shown as percentage. Group differences were assessed using Fisher’s exact test. Anemia and iron deficiency were defined according to the study’s prespecified laboratory cut-offs.

**Figure 4:**
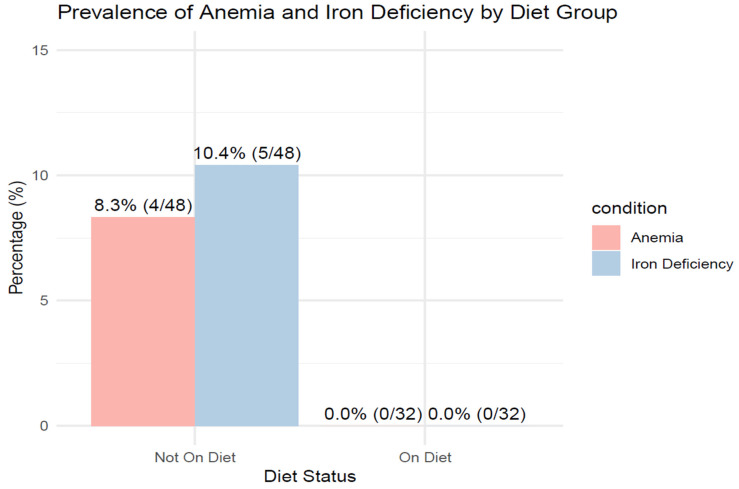
Postoperative prevalence of anemia and iron deficiency in both diet groups. Bars represent the proportion of patients with anemia and iron deficiency in each diet group. Values above bars are shown as percentage. Group differences were assessed using Fisher’s exact test. Anemia and iron deficiency were defined according to the study’s prespecified laboratory cut-offs.

In both dietary groups, a slight decrease in ferritin concentration was observed, which was not statistically significant (CI (−37.98)–(−40.59), *p* = 0.947), (Table 2); moreover, in the group that did not follow the Mediterranean diet, a deficiency of this protein was found in 3 patients before surgery (Figure 3) and 4 patients after surgery (Figure 4).

Hemoglobin levels decreased slightly in both dietary groups, but the difference between the two interventions was not statistically significant (CI (−0.61)–0.23, *p* = 0.379), (Table 2). Additionally, anemia was observed in 3 patients before surgery (Figure 3) and in 4 patients after surgery (Figure 4). None of the patients with anemia adhered to the Mediterranean diet.

### 3.4. Influence of Mediterranean Diet Adherence on EWL% Values

**Figure 5:**
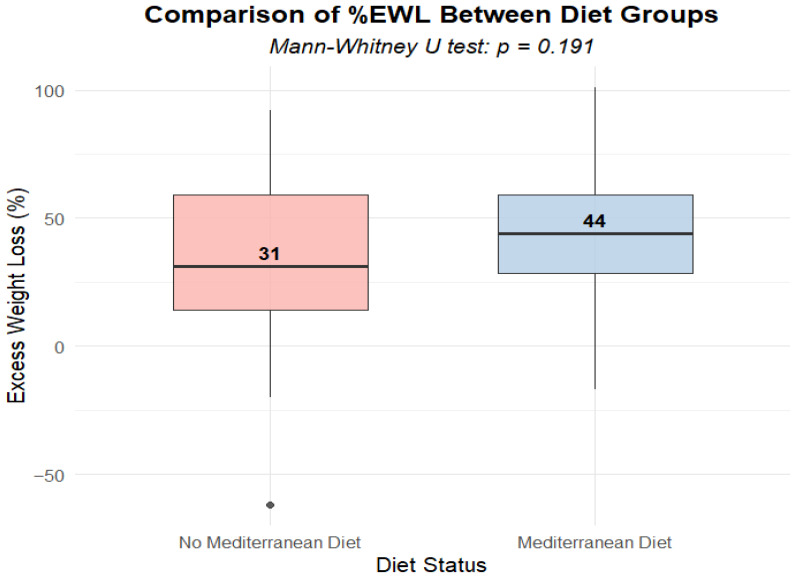
Comparison of percentage excess weight loss (%EWL) between both diet groups. Boxplots show the distribution of percentage excess weight loss (%EWL) in patients with and without adherence to the Mediterranean diet. The horizontal line represents the median, boxes indicate the interquartile range (IQR), and whiskers denote minimum and maximum values (excluding outliers). Between-group comparisons were performed using the Mann–Whitney U test.

Both the median and mean EWL% were found to be higher in patients following the Mediterranean diet (median = 44% vs. 31%, mean = 43.75 vs. 34.04). However, the difference between the two dietary interventions was not statistically significant (*p* = 0.144) (Figure 5).

### 3.5. Influence of Protein Supplementation on EWL% Values

**Figure 6:**
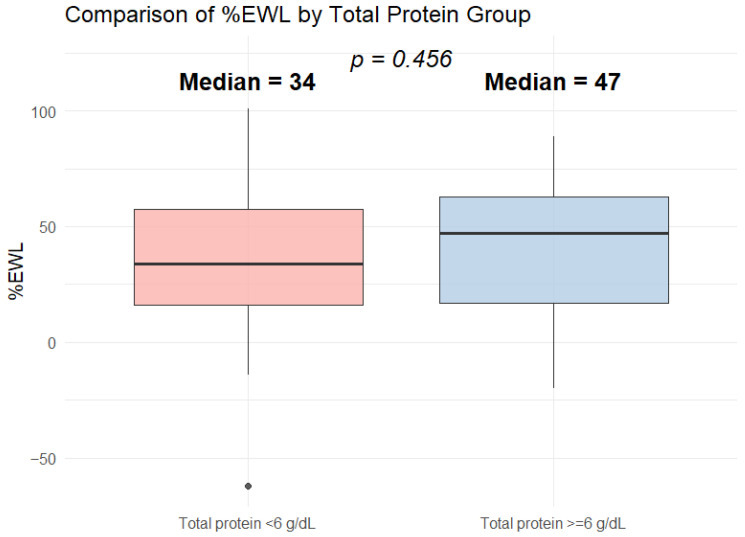
Comparison of percentage excess weight loss (%EWL) according to preoperative total protein status. Boxplots represent the distribution of percentage excess weight loss (%EWL) in patients stratified by preoperative total serum protein level (<6 g/dL and ≥6 g/dL). The horizontal line indicates the median, boxes denote the interquartile range (IQR), and whiskers represent minimum and maximum values (excluding outliers). Between-group comparisons were performed using the Mann–Whitney U test.

Patients with total protein levels greater than or equal to 6 g/dL had a higher mean EWL% than those with lower preoperative levels (mean: 40.9 vs. 36.2, median: 47% vs. 31%). Furthermore, the median EWL% in the group of 50 patients with preoperative total protein levels below the normal range was significantly lower than in the remaining patients. However, this difference was not statistically significant, and no correlation was observed between preoperative total protein levels and EWL% (*p* = 0.456) (Figure 6).

**Figure 7:**
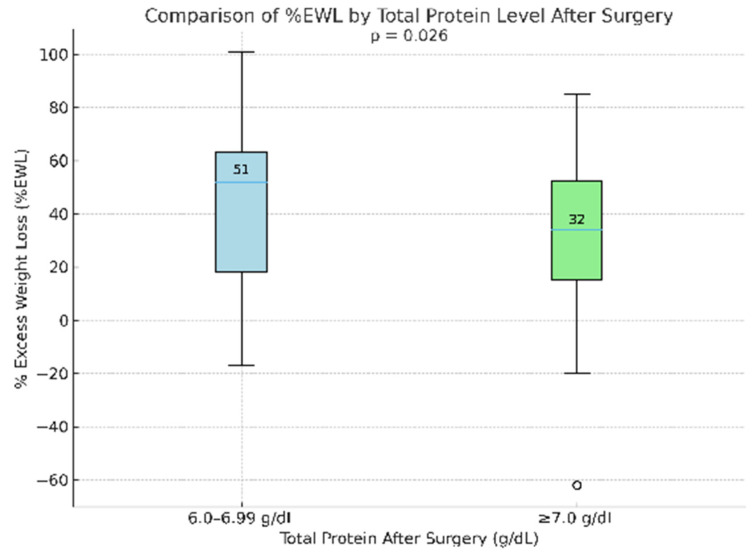
Comparison of percentage excess weight loss (%EWL) according to postoperative total serum protein level. Boxplots illustrate the distribution of percentage excess weight loss (%EWL) according to postoperative total serum protein levels (6.0–6.99 g/dL vs. ≥7.0 g/dL). The horizontal line represents the median, boxes indicate the interquartile range (IQR), and whiskers denote minimum and maximum values (excluding outliers). Between-group differences were assessed using the Mann–Whitney U test.

Postoperatively, all patients had total protein levels within the normal range of 6–8 g/dL. In the group of patients with total protein levels of 6–6.99 g/dL, the median EWL% was higher than in the group with total protein levels greater than or equal to 7 g/dL (median: 51% vs. 32%). This difference was statistically significant, although there was no correlation between postoperative total protein levels and EWL% (r = −0.16, *p* < 0.05) (Figure 7).

## 4. Discussion

Our study aimed to assess the impact of a Mediterranean diet and recommended postoperative supplementation on laboratory parameters after bariatric surgery. In the analyzed sample, despite observed improvements in individual marker concentrations after surgery, no statistically significant differences were detected between the two dietary groups (On Diet vs. Not On Diet) regarding vitamin D, vitamin B12, folic acid, calcium, and total protein. These findings may indicate that the vast majority of patients met the recommended intake of protein, micronutrients, and vitamins after surgery [7,13]. However, given the relatively small sample size and the limited number of participants adhering to the Mediterranean diet, the lack of statistical significance should be interpreted cautiously and does not necessarily imply the absence of a potential dietary effect. The effect of recommended supplementation appeared to be dominant, to the extent that distinguishing it from the independent effect of the Mediterranean diet was difficult. This is particularly true for the aforementioned parameters, where a significant increase in concentrations was observed in both dietary groups after surgery, but comparison of differences between them did not reveal statistical significance, suggesting that supplementation may have played a major role in normalization, while a potential independent dietary contribution cannot be excluded. The main limitations of our study include the limited sample size (*n* = 80), due to the inability to meet all inclusion criteria for further analysis, including complete and paired pre- and postoperative data, which could potentially reduce statistical power and the ability to detect significant associations. Within our sample, only 32 individuals who adhered to the Mediterranean diet were identified, which may have further limited statistical power. Therefore, non-significant findings should be interpreted as exploratory and hypothesis-generating rather than definitive.

An additional factor influencing the results of the Mediterranean diet after surgery is the use of recommended nutrient intakes, established centrally by international scientific societies (IFSOs). In our study, this increased the concentration of individual nutrients in both dietary groups, making it difficult to distinguish the effect of diet from the effect of supplementation. To accurately assess the benefits of the diet, we also compared the concentrations of the nutrients we studied before surgery between those who followed the Mediterranean diet and those who did not, bearing in mind that the patients did not use nutrient supplementation in the preoperative period [7,13]. However, differences in preoperative vitamin D, vitamin B12, folate, calcium, and total protein values between the two dietary groups were also not statistically significant, suggesting that other factors besides dietary patterns may have influenced the concentrations of individual parameters. Both dietary groups also had common vitamin D deficiency and decreased total protein values.

Low total protein levels may be related to obesity itself, as it is associated with chronic inflammation of adipose tissue, often accompanied by hypoalbuminemia, which contributes to reduced total protein concentrations [20]. Additionally, in a 12-week intervention aimed at assessing the effect of the Mediterranean diet, aerobic training and additional hemp protein supplementation on biochemical parameters and plasma amino acid levels in overweight or obese patients, an increase in the concentration of some amino acids, such as α-aminoadipic acid and methylhistidine and a significant improvement in the lipid profile were observed [41]. Although total protein levels were not assessed in that study, these findings suggest that interventions combining diet, exercise, and plant protein supplementation may influence markers related to protein metabolism [41]. Another study compared the short-term effects of a Mediterranean diet, a high-protein diet, and a high-protein diet supplemented with whey protein on lipid profile, body composition, and muscle damage in overweight, sedentary individuals [42]. The design of that intervention confirmed that the Mediterranean diet was characterized by a significantly lower protein intake compared with the protein—rich dietary models [42], which in our study may potentially be reflected in the lower total protein concentrations observed before surgery. Importantly, despite differences in protein intake, all dietary approaches resulted in comparable weight loss and improvements in selected lipid parameters [42]. At the same time, reduced physical activity was identified as a factor potentially attenuating metabolic benefits [42]. In our study, we did not assess the effects of physical activity, as the primary objective was to evaluate the association between Mediterranean diet adherence and selected nutrient concentrations, particularly in the postoperative setting. Furthermore, additional protein supplementation was not introduced before surgery in order to reflect standard post-bariatric clinical practice, where recommended supplementation primarily concerns the postoperative period [7,13].

Moreover, high Body Mass Index (BMI) and large adipose tissue volume are factors that may contribute to lower circulating vitamin D concentrations [43]. However, a cross-sectional study conducted in Italy, including 284 overweight and obese individuals, reported that adherence to a Mediterranean diet was associated with higher blood vitamin D levels, regardless of the Body Mass Index (BMI) [43]. It should be noted, however, that this study was conducted in the Mediterranean basin, where, in addition to diet, climate conditions characterized by high levels of sunlight exposure may have substantially influenced vitamin D status [43]. In our study, data collection was performed at different times of the year, corresponding to varying levels of solar radiation exposure. Given the well—established impact of sunlight on endogenous vitamin D synthesis, seasonal variability may have influenced the measured concentrations of this marker and potentially contributed to variability in the observed results. Furthermore, a population-based study including over 500,000 individuals living in Catalonia found that 75% of participants had suboptimal vitamin D levels despite residing in a Mediterranean region [44]. Notably, higher concentrations of this parameter were observed only in individuals receiving additional vitamin D supplementation [44]. In the preoperative period, patients in our cohort did not use vitamin D supplementation, which underscores the importance of postoperative supplementation. At the same time, given the relatively small sample size and limited statistical power of our study, the absence of statistically significant differences between dietary groups should be interpreted with caution and viewed as exploratory rather than conclusive [7,11,13,44].

Preoperative values for other nutrients in both dietary groups were within reference ranges, indicating that all patients, regardless of whether they followed a Mediterranean diet, had normal levels of vitamin B12, folate, and calcium. Nevertheless, current medical literature suggests potential beneficial associations between this dietary pattern and selected micronutrient levels; however, in our cohort, no statistically significant differences between groups were observed, and these findings should therefore be interpreted cautiously, particularly in light of the relatively small sample size.

In a population-based analysis of women from southern Spain, higher scores on the Mediterranean Diet Score (MDS), a tool measuring adherence to the Mediterranean diet, were associated with higher folate intake and a lower likelihood of deficiency, regardless of age, education, or place of residence [45]. Similar associations were reported in the IMMEDIATE study, which included populations from Italy and Great Britain [46]. High intake of products typical of the Mediterranean diet was associated with higher serum folate concentrations, with each 100 μg/day increase in folate intake resulting in a 13.8% increase in serum folate concentration in the Italian population and a 10.5% increase in the British population [46]. Furthermore, a study of pregnant women from southern Italy demonstrated that low adherence to the Mediterranean diet was significantly associated with a higher likelihood of insufficient folate intake [47]. In the subsample of women who completed their pregnancy, the rate of SGA (Small for Gestational Age) was higher in women with insufficient folate intake (*p* < 0.001) [47]. In a cohort study from Barcelona, including elderly individuals at high cardiovascular risk, adherence to the Mediterranean diet was associated with higher folate concentrations, whereas vitamin B12 concentrations did not differ significantly between groups [48]. Although individuals with higher vitamin B12 levels demonstrated better memory performance, this association was observed among those following a Mediterranean diet and does not necessarily imply a direct effect of the dietary pattern on vitamin B12 concentrations [48]. A study that showed similar results was a cross-sectional analysis conducted in the Netherlands that compared the association between dietary sources of vitamin B12, including meat and meat products, dairy products, fish and seafood, and eggs, and serum vitamin B12 concentrations in older participants [49]. It was found that higher consumption of dairy products, meat, fish or seafood was significantly associated with higher serum vitamin B12 levels, while egg consumption did not cause a significant increase in this parameter [49]. Based on these findings, it may be inferred that dietary patterns with higher intake of meat and dairy products are associated with higher serum vitamin B12 concentrations, compared with dietary patterns characterized by more moderate amounts of these products, such as the Mediterranean diet; however, it should be emphasized that not all individuals with lower meat consumption were vitamin B12 deficient [49,50]. Therefore, although the Mediterranean diet may not significantly increase the serum vitamin B12 concentrations, available evidence suggests it can support maintaining adequate vitamin B12 status in many individuals [48,49]. Although in our study there were no statistically significant differences in folic acid or vitamin B12 concentrations between patients following the Mediterranean diet and those who did not, all participants adhering to the Mediterranean diet did not present deficiencies of these vitamins, which is in line with the current medical literature [45,46,47,48]. Importantly, given the relatively small sample size, especially within the Mediterranean diet, adherent subgroup and the resulting limited statistical power, these non-significant findings should be interpreted cautiously as exploratory and hypothesis-generating rather than definitive evidence of no association. Consequently, our results should not be interpreted as excluding a potential relationship between Mediterranean diet adherence and these micronutrient parameters, and further adequately powered studies are warranted to clarify these associations.

In our study, no statistically significant difference was observed between the two preoperative dietary groups, including for calcium levels. However, the current literature demonstrates varying relationships between adherence to the Mediterranean diet and calcium levels or intake. In a large observational study examining the relationship between the DASH (Dietary Approaches to Stop Hypertension) diet, which focuses on lowering blood pressure, and the Mediterranean diet in older patients with age-related cognitive changes, no significant differences in calcium intake were observed between quintiles of the Mediterranean diet score. In contrast, the DASH diet score showed more pronounced differences in calcium intake [51]. The DASH diet results demonstrated a significant, increasing relationship with calcium intake, suggesting that the higher adherence scores were associated with greater calcium intake, whereas the Mediterranean diet showed a non-significant relationship with calcium intake [51]. In turn, an observational survey conducted among 279 pregnant women from Florence, assessing the effect of adherence to the Mediterranean diet on calcium intake, showed that higher adherence was associated with higher calcium intake [52]. Although this study suggests that the Mediterranean diet may promote higher intake of certain nutrients, the average calcium intake in that intervention remained below Italian recommendations for pregnant women [52]. Another cohort study conducted in Sweden, including 82,000 adult participants, examining the association of dietary calcium intake, combined with adherence to the Mediterranean diet and the risk of hip fracture, showed that adherence to the Mediterranean diet together with calcium intake above 800 mg/day was associated with the lowest risk of hip fracture, whereas low adherence was associated with a higher fracture risk even at high calcium intake [53]. These findings suggest that the Mediterranean diet may play a supportive role in overall dietary patterns, although its independent effect on increasing calcium levels appears limited [51,52,53]. The results of our study are consistent with the current literature, as the differences in preoperative calcium levels between the diet and non-diet groups were not statistically significant. However, given the relatively small sample size, particularly in the subgroup adhering to the Mediterranean diet, these non-significant findings should be interpreted cautiously and considered exploratory rather than conclusive evidence of no association. Calcium deficiency was not observed in the diet group, although absolute calcium values were not high, which may reflect the moderate influence of the Mediterranean diet on this parameter [51]. Moreover, our study sample did not include pregnant women, and pregnancy is a state of increased calcium demand [52,54]. Therefore, the calcium intake threshold in our cohort was lower, and it is possible that dietary intake was sufficient to prevent calcium deficiency under these conditions. Additionally, in the preoperative period, patients did not use calcium supplementation, which would translate into an increased supply of this nutrient; thus, the supportive effect of diet during this period could not be fully assessed [7,13,53]. It is possible that such an effect may have been present in the postoperative period; however, its independent contribution is difficult to determine due to the predominant effect of recommended calcium supplementation [7,13].

The only statistically significant difference between the two dietary groups, both before and after surgery, was iron concentration (*p* < 0.05). While this finding may suggest a potential association between adherence to the Mediterranean diet and iron metabolism, it should be interpreted with caution, particularly given the relatively small number of iron deficiency cases in our cohort. Furthermore, iron concentrations were within the reference range for all patients following the Mediterranean diet both before and after surgery, whereas in the group not following this dietary pattern, iron deficiency was observed in three patients before surgery and in five patients after surgery. A possible cause of preoperative iron deficiency could be chronic inflammation associated with obesity, which, as indicated in the literature, may increase the production of hepcidin, a positive acute phase protein, leading to reduced iron uptake in the intestine and its excessive accumulation in adipose tissue [55]. Some studies suggest that after bariatric surgery, there is a gradual reduction in inflammation, which may promote improved iron metabolism [55]. However, due to the lack of inflammatory markers such as CRP both before and after bariatric surgery, these mechanisms remain hypothetical in the context of our study and cannot be considered a direct explanation of the observed findings. The occurrence of iron deficiency after surgery may also reflect insufficient coverage by recommended supplementation or suboptimal adherence to supplementation guidelines. Poor adherence to iron recommendations has been reported in a cohort study assessing adherence to vitamin and mineral supplementation after bariatric surgery [56]. Adherence to iron therapy after surgery was shown to be significantly lower than with other supplements and may have contributed to the increased incidence of anemia [56]. Another observational study, which also examined patients’ adherence to postoperative recommendations and reasons for non-compliance, demonstrated that a substantial proportion of patients did not take all recommended supplements, used them irregularly, or discontinued them in the long term [57]. The most commonly reported barriers included forgetfulness, high costs of therapy and side effects such as abdominal pain, vomiting, diarrhea, headaches or constipation—especially in the case of iron preparations [57]. These findings indicate that intolerance to supplements, including iron, may contribute to reduced adherence and, consequently, to the development of deficiencies [55,57]. Therefore, we speculate that postoperative iron deficiency in our study may have resulted from incomplete adherence to recommended iron supplementation; however, this explanation should be interpreted cautiously, as compliance with iron supplementation was not objectively measured and only a small proportion of our sample was iron deficient, while concentrations of other nutrients remained within their reference ranges after surgery. An additional factor that could influence the presence of pre- and postoperative iron deficiency in the group not following the Mediterranean diet, especially in female patients, could be menstrual bleeding, which may lead to a further reduction in iron stores [58]. A prospective observational study of 105 women showed a strong association between menstrual blood loss and iron loss, indicating that menstruation may be an important factor contributing to iron deficiency [58]. Furthermore, although no statistically significant differences were found in ferritin and hemoglobin concentrations between the two dietary groups, all patients on the Mediterranean diet had normal values for these parameters both before and after surgery. However, three patients with preoperative iron deficiency also had iron deficiency anemia, and of the five patients with postoperative iron deficiency, four also had anemia. The fact that neither iron deficiency nor iron deficiency anemia was observed in patients following the Mediterranean diet may suggest that this dietary pattern may have a preventive effect on iron metabolism disorders. The results of our study appear to be consistent with the current medical literature on this topic. In a cross-sectional, population-based study assessing the impact of following the Mediterranean diet on iron-related parameters in pregnant women, higher adherence to the diet was associated with higher iron intake and a lower risk of iron deficiency in the first trimester, as well as with higher red blood cell counts [59]. However, no statistically significant differences in ferritin and hemoglobin concentrations were observed between groups with different levels of adherence to the diet, although values in women with higher adherence remained within the reference ranges [59]. In a one-year randomized controlled trial involving 1294 elderly individuals from different European countries, which aimed to assess the impact of the Mediterranean diet on inflammatory responses and nutritional status, it was found that although participants following the diet demonstrated increased in iron intake and iron deficiency was rare in this group, no overall statistically significant change in iron status was observed in the combined analysis across all countries [60]. At the same time, the authors suggested that the Mediterranean diet may provide sufficient amounts of bioavailable iron, even with lower meat consumption, which may help maintain normal iron status [60]. Additionally, a four-year prospective study including patients with obesity and compensated cirrhosis after bariatric surgery who underwent an intervention based on the Mediterranean diet demonstrated a statistically significant improvement in anthropometric parameters and selected hematological markers [61]. Analysis of the Supplementary Data indicated a statistically significant increase in ferritin and hemoglobin concentrations during the observation period [61]. Although the study did not directly assess iron metabolism, the reported improvements in hematological parameters, including ferritin and hemoglobin, may suggest a potentially beneficial association, but they do not establish a definitive causal relationship [61]. However, it should be emphasized that due to the complex clinical profile of the studied population and the role of ferritin as an acute phase protein [39], interpretation of these findings requires caution [61]. In the context of our findings, adherence to the Mediterranean diet may be associated with higher iron intake, which could potentially translate into more favorable iron-related parameters and a reduced risk of iron deficiency. Nevertheless, these observations should be interpreted carefully, particularly in light of incomplete adherence to recommended supplementation in patients after bariatric surgery and the relatively limited statistical power of our analysis. Furthermore, although the diet may contribute to maintaining ferritin and hemoglobin within reference ranges, especially in the postoperative setting, these findings should be regarded as indicative of possible trends rather than conclusive evidence of a protective effect against anemia.

In our study, patients declaring adherence to a Mediterranean diet achieved a higher EWL% than those not following the diet (median: 44% vs. 31%, mean: 43.75% vs. 34.04%); however, the differences between the dietary groups were not statistically significant (*p* = 0.191). The findings may indicate a trend toward a more favorable reduction in excess body weight among patients adhering to the Mediterranean diet. Nevertheless, considering the relatively small sample size (*n* = 80), particularly the limited number of participants in the Mediterranean diet subgroup (On Diet = 32), as well as the variability in EWL% values, the study may have lacked sufficient statistical power to detect moderate between—group differences. Therefore, the absence of statistical significance should be interpreted cautiously and not as definitive evidence of no effect. The lack of statistical significance may also be related to potential confounding factors, such as varying levels of physical activity or the impact of overlapping postoperative interventions, including recommended protein supplementation [41,42]. Importantly, the direction of the observed associations is consistent with findings reported in the literature, in which greater adherence to the Mediterranean diet was associated with improved weight loss outcomes. A systematic review of studies on predictors of surgical treatment outcomes for obesity, with particular emphasis on weight loss, demonstrated that adherence to dietary recommendations, including regular meal consumption, avoidance of excessively high-calorie foods, and adequate protein intake, was a significant predictor of weight loss [62]. Moreover, these studies highlighted that no single individual factor, such as Body Mass Index (BMI), age or type of surgery, had as strong an association with weight loss as adherence to dietary recommendations [62]. Although this review did not explicitly refer to adherence to the Mediterranean diet per se, it supports the broader conclusion that appropriate dietary behavior is a key determinant of postoperative weight loss [62]. Additionally, a prospective observational study, evaluating whether diet or physical activity mediates weight loss after bariatric surgery, found that individuals who increased adherence to the Mediterranean diet achieved significantly higher %Total Weight Loss (TWL%) after one year compared to those whose adherence decreased [63]. In the case of physical activity, there were no statistically significant differences in body weight changes between people who increased their physical activity and those who maintained or decreased it [63]. Additionally, in a prospective observational study of patients undergoing laparoscopic sleeve gastrectomy (LSG), the aim of which was to assess adherence to the Mediterranean diet, as measured by the KIDMED index, before and 1 year after surgery, a higher score of this index was found to be correlated with greater weight loss and a more favorable lipid profile [64]. These findings indicate an association between higher adherence to the Mediterranean diet and weight loss; however, given the observational design of these studies, causal inference remains limited. Moreover, the PREDIMED-Plus randomized clinical trial, including overweight or obese adults from Spanish research centers, evaluated a 3-year intervention based on a Mediterranean diet with reduced energy intake and increased physical activity and demonstrated changes in body composition. Participants who underwent the intervention showed a significantly reduced percentage of total fat mass and, additionally, a lower loss of free fat mass (FFM), which is an important determinant of weight loss quality, as it promotes a better resting metabolic rate (RMR) and reduces the risk of sarcopenia in older age [65,66,67]. These results suggest that the Mediterranean diet, particularly when combined with regular physical exercise, may be associated not only with weight loss but also with preservation of its quality, promoting greater reduction in body fat while maintaining relative fat-free mass, including skeletal muscle [65]. However, extrapolation of these findings to our cohort should be made with caution, as our study was not designed to directly assess body composition changes or to evaluate the independent effect of diet beyond standard postoperative recommendations.

After surgery, all patients were advised to follow a recommended protein intake of 60–80 g per day or 1–1.5 g/kg ideal body weight (IBW) with an additional 30 g of supplementation, in accordance with current guidelines [8,13]. To provide an approximate assessment of protein status, total protein concentrations were monitored before and after surgery, consistent with routine clinical practice at our center. Postoperatively, total protein levels remained within the reference range in all participants. Before surgery, however, the majority of patients presented with lower values, which may reflect the absence of specific preoperative protein supplementation requirements under current guidelines [7,13].

It should be emphasized that, as reported in the current literature, total protein concentration alone does not reliably reflect actual protein intake nor exclude protein malnutrition [68]. Although total protein and albumin levels are frequently used in clinical settings as general indicators of nutritional status and adherence to dietary recommendations [19], contemporary guidelines caution against interpreting these parameters as definitive markers of adequate protein nutrition after bariatric surgery [68,69]. Their concentrations may be influenced by several factors, including inflammation, infection, hydration status and liver function [69]. Albumin, which constitutes the major fraction of total protein, is a negative acute-phase reactant and may decrease in the presence of systemic inflammation. Given that obesity is characterized by chronic low-grade inflammation [20,70], the reduced preoperative total protein levels observed in our cohort may partially reflect inflammatory status rather than insufficient protein intake per se. This interpretation is supported by a prospective cohort study of patients undergoing laparoscopic sleeve gastrectomy, which demonstrated a negative correlation between CRP and albumin concentrations before surgery [20]. However, the decrease in CRP values 12 months postoperatively did not significantly influence albumin levels (*p* = 0.309), suggesting a complex relationship between inflammatory status and protein markers [20]. Additionally, retrospective analyses have reported lower albumin concentrations six months after bariatric surgery, even in the absence of objectively confirmed protein deficiency [71]. In another intervention examining vitamin D, vitamin B12 and albumin levels 2 years after surgery, patients who reported adequate protein intake after surgery were more likely to present albumin concentrations within the reference range compared with those not adhering to recommendations [72]. These observations suggest a potential association between dietary protein intake and selected biochemical markers; however, albumin or total protein concentration alone cannot be considered a reliable indicator of protein nutritional status. Although insufficient protein supply after surgery may be reflected in reductions in these laboratory parameters [20,69,71,72], such changes should be interpreted cautiously, as they may also be influenced by inflammation, hydration status, liver function, or other clinical factors. Moreover, hypoalbuminemia and peripheral edema have been described as clinical components of protein malnutrition [37]; however, these features were not observed in our cohort. All patients had total protein values within the reference range 6 months after surgery and showed no peripheral edema or other overt clinical signs of malnutrition [38]. Therefore, while these findings may be consistent with adequate postoperative protein intake and adherence to supplementation recommendations, total protein concentration alone does not allow for definitive conclusions regarding protein nutritional status. Before surgery, the majority of participants presented lower total protein levels, which could potentially reflect chronic low-grade inflammation associated with obesity rather than true protein deficiency [7,13,20,69]. To more comprehensively exclude postoperative malnutrition, additional objective measures such as free fat mass assessment and muscle strength evaluation would be required [68,73]. Our study did not include body composition assessment, as the primary objective was to explore potential associations between postoperative protein intake, total protein concentrations and excess weight loss expressed as EWL%, in light of the limited literature addressing this relationship [19]. To obtain a more objective evaluation of compliance with postoperative protein recommendations, additional methods such as 3-day food diaries or repeated 24-h dietary recalls could be considered in future studies to allow for a more precise estimation of daily protein intake from both diet and supplementation [74,75].

In our cohort, patients with preoperative total protein concentrations near the lower limit of normal tended to present lower EWL% values compared with those with values within the reference range (mean EWL%: 36.2 vs. 40.9). However, this difference did not reach statistical significance (*p* = 0.456), and no correlation was observed between preoperative total protein concentration and EWL% (r = −0.16). The lack of statistical significance may reflect limited statistical power due to the relatively small sample size (*n* = 80), as only patients with complete paired pre- and postoperative data were included in this analysis. Therefore, these findings should be interpreted with caution and cannot exclude the possibility of type II error. Furthermore, preoperative total protein levels may be influenced by chronic low-grade inflammation associated with obesity [20,76], which complicates the interpretation of their relationship with postoperative weight loss. Previous studies have shown that higher values of the Systemic Inflammatory Response Index (SIRI), defined as the product of neutrophil and monocyte counts divided by the lymphocyte count, were associated with greater obesity severity and lower EWL% after bariatric surgery [77]. Similarly, elevated preoperative Neutrophil-to-Lymphocyte Ratio (NLR) has been linked with less favorable postoperative weight outcomes [70]. Therefore, the lack of correlation between preoperative total protein concentration and EWL%, as well as the absence of a statistically significant difference, may suggest that adequate preoperative protein intake is not independently associated with postoperative weight loss within the limits of this study design. At the same time, the observed trend toward higher EWL% values in patients with normal preoperative total protein levels warrants further investigation in larger cohorts and with more comprehensive preoperative assessments, including inflammatory markers such as CRP and direct measures of body composition, e.g., free fat mass (FFM), to better characterize protein nutritional status.

Six months after surgery, we observed that patients with total protein concentrations between 6 and 6.99 g/dL had higher EWL% values than those with values above 7 g/dL (median: 51% vs. 32%). Although no correlation was found between EWL% and total protein concentration after surgery, the difference between the two groups reached statistical significant (r = −0.16, *p* < 0.05). Importantly, all total protein values remained within the reference range and patients adhered to postoperative dietary recommendations, including recommended protein supplementation [7,8,13]. While adequate total protein levels may reflect sufficient protein intake, total protein concentration represents an indirect and imperfect marker of protein nutritional status and may be influenced by inflammatory status, hydration, and other non-nutritional factors. The absence of a linear association between total protein concentration and EWL% suggests that postoperative weight loss is likely multifactorial and cannot be explained by protein intake alone. The significant intergroup difference observed in the absence of correlation may indicate a complex or potentially non-linear relationship, or the influence of additional confounding variables not directly related to protein intake. One possible explanation may involve persistent low-grade inflammation despite surgical intervention, particularly in patients with higher total protein levels, although this remains speculative and cannot be confirmed within the scope of present study [70,77]. Another potential factor could be postoperative dehydration, a common occurrence after bariatric surgery, which may artificially increase totalprotein concentrations in some individuals [21]. Furthermore, a randomized trial evaluating the effect of protein supplementation 1, 3, and 6 months after laparoscopic sleeve gastrectomy showed that although recommended protein intake and supplementation improved total protein concentration after surgery, they did not significantly affect weight loss [14]. Based on our results and the current medical literature, our findings do not support a clear association between higher protein intake and greater weight loss, as measured by EWL%. However, these observations should be interpreted with caution, given the indirect nature of total protein as a marker of protein nutritional status and the observational design of the study. Some studies indicate that short-term high-protein diets, including those enriched with additional whey protein supplementation, may promote weight loss. Nevertheless, their reported effects appear comparable to those observed with the Mediterranean diet, which contains a relatively lower protein content. These data suggest that higher protein intake alone may not be the sole determinant of postoperative weight reduction [42]. Moreover, the literature indicates that free fat mass (FFM) is one of the key determinants of resting metabolic rate (RMR) and may influence the rate and extent of body weight reduction [66]. The absence of direct body composition assessment, particularly free fat mass (FFM), represents an important limitation of the present study and precludes a definitive evaluation of whether the observed differences in EWL% are related to the patients’ functional and metabolic status. Future studies should consider incorporating direct measures of body composition, along with detailed assessments of protein intake and markers of hydration and inflammation, to better characterize the complex relationships between protein status and postoperative weight loss after bariatric surgery.

Further prospective studies are warranted to clarify the associations between Mediterranean diet adherence, postoperative nutritional status (vitamin D, vitamin B12, folic acid, total protein, and calcium), excess weight loss (EWL%), and the role of protein nutritional status in postoperative weight loss outcomes.

### Limitations of This Study

The main limitation of our study was the small sample size (*n* = 80), due to the lack of complete and paired pre- and postoperative data for nineteen patients, which could have potentially reduced the statistical power of the tests and the ability to detect significant associations. Furthermore, the reduced number of participants may have contributed to the uneven distribution of sample sizes between the dietary groups. The Mediterranean diet group included 32 patients, whereas the non-Mediterranean diet group included 48. This imbalance may have further contributed to the reduced statistical power and made it difficult to detect subtle differences between the groups. Furthermore, assessing the effect of the Mediterranean diet on individual laboratory parameters after surgery was hampered by the dominant effect of the recommended postbariatric supplementation. It should also be noted that the method of dietary assessment is one of the limitations of this study. Data on dietary habits were collected as part of a broader clinical assessment of patients, and diet was not initially the primary focus of the research project. Consequently, standardized tools for assessing adherence to the Mediterranean diet, such as the MEDAS or KIDMED questionnaires, were not used, which may have limited the accuracy of assessing patients’ actual compliance with this dietary pattern. Moreover, no quantitative assessment of dietary nutrient intake was performer, which limits precise evaluation of micronutrient exposure and comparability with studies using standardized dietary scoring systems. Nevertheless, the adopted approach allowed for a practical assessment of patients’ actual dietary habits in a clinical setting, which was consistent with the observational nature of the study. Another potential limitation may have been the failure to consider the effect of physical activity on weight loss and laboratory parameters, primarily protein. Additionally, our study did not include tools such as food diaries, which would have facilitated accurate patient adherence to dietary and supplementation recommendations. Another limitation may have been the lack of postoperative assessment of inflammatory markers such as C-reactive protein (CRP), which would have allowed a more accurate interpretation of total protein concentrations, given that inflammation may influence circulating protein levels and potentially confound their relationship with weight loss. Similarly, the absence of postoperative hydration assessment represents another important limitation, as a change in fluid balance after bariatric surgery may affect total protein concentrations independently of actual protein nutritional status. Furthermore, our study did not include postoperative assessment of free fat mass (FFM), which constitutes a more direct and clinically relevant indicator of protein nutritional status and metabolic function. The lack of body composition analysis limits the ability to determine whether observed differences in EWL% were related to variations in functional protein reserves or metabolic capacity. Therefore, the interpretation of total protein as a marker of protein nutritional status in this study should be approached with caution and the observed associations should be considered exploratory rather than definitive evidence of a causal relationship.

Given the observational design of the study and the non-random allocation of participants to dietary patterns, causal inference is inherently limited. The observed relationships between the Mediterranean diet adherence and selected laboratory parameters, including iron status, should therefore be interpreted as associations rather than evidence of direct causal effects. Residual confounding, unmeasured lifestyle factors, and differences in adherence to supplementation may have contributed to the reported findings. Moreover, the use of standardized postoperative supplementation in all patients may have attenuated potential differences attributable to dietary patterns. Although multivariable adjustment was performed for outcomes demonstrating significant between-group differences, residual confounding due to unmeasured factors (e.g., comorbidities or physical activity) cannot be excluded. Because multivariable analyses were restricted to outcomes with significant unadjusted differences, some associations may have remained undetected due to residual or counteracting confounding. Therefore, the findings should be interpreted with appropriate caution. Furthermore, multiple statistical comparisons were performed without formal correction for multiplicity, which may increase the risk of type I error. In addition, the relatively small number of patients with iron deficiency, the lack of objective assessment of adherence to recommended iron supplementation, and the absence of quantitative evaluation of dietary iron intake limit the strength of iron-related conclusions and warrant cautions interpretation of these findings. Additionally, the 6-month follow-up period limits the ability to assess long-term nutritional deficiencies and sustained weight-loss trajectories, which may evolve over 12–24 months after bariatric surgery.

## 5. Conclusions

A Mediterranean dietary pattern, when combined with recommended postoperative supplementation, was associated with a numerically lower prevalence of iron deficiency and anemia before and after bariatric surgery in our cohort. However, given the relatively small number of deficiency cases and the lack of objective assessment of adherence to iron supplementation and quantitative evaluation of dietary iron intake, these findings should be interpreted with caution. Adherence to a Mediterranean diet, together with recommended postoperative protein intake, was also associated with a tendency toward greater weight loss expressed as EWL%; however, these differences did not reach statistical significance and may have been influenced by limited statistical power and sample size. Furthermore, total protein concentration assessed before and after surgery may represent an imperfect marker of protein nutritional status, as it can be affected by factors independent of dietary protein intake, including inflammation and hydration status. Overall, the findings of this observational study suggest possible associations between dietary patterns and selected postoperative outcomes; however, causal inferences cannot be made. Larger, adequately powered prospective studies with comprehensive assessment of dietary intake, supplementation adherence, and body composition are required to confirm these observations and clarify underlying mechanisms.

## Figures and Tables

**Table 1:** Changes in selected nutritional parameters before and after bariatric surgery stratified by Mediterranean diet adherence. Data are presented as median and interquartile range (IQR). Pre- and postoperative comparisons were performed using the Wilcoxon signed-rank test. Statistical significance was set at *p* < 0.05. N—number of patients in each subgroup; *—*p* < 0.05; **—*p* < 0.01; ***—*p* < 0.001.

Parameter	N	Diet Status	Before (Median)	Before (IQR)	After (Median)	After (IQR)	Median Diff	*p*-Value	Significance
Vitamin D	48	Not on Diet	23.85	18.62–26.83	32.8	27.62–40.18	8.95	0.0000	***
	32	On Diet	22.25	18.55–25.60	32.55	26.68–36.23	10.3	0.0000	***
	80	Overall	23.35	18.55–26.80	32.8	26.87–37.00	9.45	0.0000	***
Folic Acid	48	Not on Diet	8.55	5.88–13.47	9.45	6.80–13.90	0.9	0.0143	*
	32	On Diet	8.35	5.80–10.85	10.05	8.38–14.23	1.7	0.0047	**
	80	Overall	8.4	5.80–12.90	9.75	7.38–14.12	1.35	0.0002	***
Vitamin B12	48	Not on Diet	447.4	309.97–492.27	441.1	345.02–532.20	−6.3	0.1880	
	32	On Diet	451.75	361.18–482.65	468.2	376.95–532.93	16.45	0.0308	*
	80	Overall	451.4	318.35–487.85	452.25	348.40–532.20	0.85	0.0132	*
Iron	48	Not on Diet	94.5	80.00–106.75	90.5	80.00–120.50	−4	0.3800	
	32	On Diet	93	73.75–108.50	117	109.50–138.50	24	0.0001	***
	80	Overall	94.5	78.50–108.25	109	88.75–130.25	14.5	0.0005	***
Calcium	48	Not on Diet	9.6	9.30–9.80	9.7	9.38–9.90	0.1	0.2610	
	32	On Diet	9.6	9.38–9.72	9.8	9.60–9.90	0.2	0.0087	**
	80	Overall	9.6	9.30–9.80	9.8	9.50–9.90	0.2	0.0122	*
Total Protein	48	Not on Diet	5.85	5.60–6.30	7	6.70–7.20	1.15	0.0000	***
	32	On Diet	5.9	5.60–6.20	7	6.70–7.20	1.1	0.0000	***
	80	Overall	5.9	5.60–6.30	7	6.70–7.20	1.1	0.0000	***
Ferritin	48	Not on Diet	68.5	41.50–120.00	77.5	39.75–114.50	9	0.8530	
	32	On Diet	63.5	37.00–129.00	68	45.75–128.75	4.5	0.6070	
	80	Overall	66	38.00–125.25	72	40.00–119.25	6	0.8600	
Hemoglobin	48	Not on Diet	13.8	13.17–14.72	13.45	12.80–14.50	−0.35	0.0270	*
	32	On Diet	14	13.55–15.03	14.25	13.40–14.70	0.25	0.2170	
	80	Overall	13.9	13.38–14.90	13.6	12.90–14.70	−0.3	0.0117	*

**Table 2:** Comparison of mean pre—to postoperative changes in nutritional parameters between patients adhering and not adhering to the Mediterranean diet. Data are presented as mean ± standard deviation (SD). Mean differences represent changes between preoperative and postoperative values. Between-group comparisons were performed using the independent samples *t*-test. Statistical significance was set at *p* < 0.05.

Parameter	Mean (SD) Difference on Diet	Mean (SD) Difference Not on Diet	t-Value	*p*-Value	95% CI Low	95% CI High
Total Serum Protein	0.88 (0.57)	0.99 (0.54)	0.869	0.388	−0.14	0.36
Total Serum Calcium	0.17 (0.34)	0.05 (0.4)	−1.482	0.143	−0.29	0.04
Ferritin	−4.47 (93.79)	−3.17 (72.44)	0.066	0.947	−37.98	40.59
Vitamin D	10.36 (6.46)	10.02 (10.01)	−0.183	0.855	−4	3.33
Vitamin B12	52.08 (116.85)	27.92 (155.55)	−0.792	0.431	−84.91	36.59
Folic Acid	1.67 (4.16)	1.29 (4.32)	−0.395	0.694	−2.31	1.54
Hemoglobin	−0.19 (0.78)	−0.38 (1.11)	−0.885	0.379	−0.61	0.23
Iron	28.66 (36.19)	5.85 (34.76)	−2.805	0.007	−39.04	−6.56

## Data Availability

The datasets generated and analyzed during the current study are available from the corresponding author upon reasonable request, subject to restrictions related to patient confidentiality and data protection regulations.

## Abbreviations

The following abbreviations are used in this manuscript:

BMI	Body Mass Index
EWL%	percentage of excess weight loss
FFMF	Free Fatty Mass
CRPC	reactive protein
RMR	Resting Metabolic Rate
NLR	Neutrophil-Lymphocyte Ratio
SIRI	Systemic Inflammatory Response Index
IBW	Ideal Body Weight
TWL	Total Weight Loss
SD	Standard Deviation
DASH	Dietary Approaches to Stop Hypertension
SGA	Small for Gestational Age
IFSO	International Federation for the Surgery of Obesity and Metabolic Disorders
IQR	Interquartile Range
MDS	Mediterranean Diet Score
IDA	Iron Deficiency Anemia
Q3	Upper quartile
CI	Confidence Interval
D3	Vitamin D3
B12	Vitamin B12
LSG	Laparoscopic Sleeve Gastrectomy
LRYGB	Laparoscopic Roux-en-Y gastric bypass
IU	International Units
